# The assessment of the match performance of association football referees: Identification of key variables

**DOI:** 10.1371/journal.pone.0291917

**Published:** 2023-09-21

**Authors:** Vítor Carvalho, Pedro T. Esteves, Célia Nunes, Werner F. Helsen, Bruno Travassos

**Affiliations:** 1 Department of Sport Sciences, Universidade da Beira Interior, Covilhã, Portugal; 2 Polytechnic Institute of Guarda, Guarda, Portugal; 3 Research Center in Sports Sciences, Health Sciences and Human Development, CIDESD, Vila Real, Portugal; 4 Department of Mathematics and Center of Mathematics and Applications, Universidade da Beira Interior, Covilhã, Portugal; 5 Department of Movement Sciences, Research Group for Movement Control and Neuroplasticity, KU Leuven, Leuven, Belgium; 6 Portugal Football School, Portuguese Football Federation, FPF, Oeiras, Portugal; Universiti Malaysia Terengganu, MALAYSIA

## Abstract

The purpose of this study was to understand the contribution of each performance indicator to evaluate match performance of football referees. Thirty-four elite Referee Match Observers (RMOs) from the Portuguese FA participated voluntarily in the study. From the official assessment sheet of each game, the referee’s game score was categorized in two groups according to referee’s game score: i) Referees with a Low Score (LFS) and ii) Referees with High Score (HFS). A multivariable binary logistic regression model was used to assess the relationship between the Performance Indicators (PIs) of the dimensions i) game difficulty and game intervention and ii) disciplinary actions and game management in relation to the referee’s match assessment score. The model proposed revealed that only the PIs negative application of the laws of the game and referee teamwork, from the dimension disciplinary actions and game management, significantly defined the final game referee’s assessment score. This study suggests that the PIs scored by the RMOs contributed in a different way to the referee’s game score. The model explains 60.4% of the referee’s game score based on the variables referee teamwork and negative application of the laws of the game.

## Introduction

In football, the referee and assistant referees (ARs) aim to apply the laws of the game according to the game demands [1]. In fact, football referees do not only need to make accurate decisions. They also need to apply proper disciplinary actions on players according to the laws of the game [1, 2], and to preserve the integrity of the game [3]. It was observed that referees make approximately 200 to 250 visible decisions per match, i.e. decisions that are observable on video footage [4]. From the total number of decisions, 28% are related with Law 12 (Fouls and misconduct), and about 31% with Law 15 (throw-in). Almost all of these decisions are made in dynamic environments, in a short period of time, in cooperation with the ARs, and with reduced visual information available and, sometimes, with the need to depict players’ deceptive movements [5]. Thus, the selection of different perceptual-action strategies to manage the game are constant, allowing the best referees to constantly pick up different information and change the focus of attention, the use of peripheral vision or the abstraction to the contextual or situational constraints [6]. In fact, high level referees tend to reveal better visual skills than the lower level ones [7].

In addition, refereeing not only implies the judgement and decision-making but also the management of the interpersonal relations with the players and other football agents to guarantee an appropriate management of the players and the game [8]. Thus, referees’ expertise is highly dependent on the consistency in decisions and management of the overall game [9]. Furthermore, elite referees show better consistency in their decision making over the games, even when they follow “unwritten rules” to support their decisions and manage the interventions according to the requirements of each game [9, 10]. Consequently, players, coaches and other agents reported high levels of satisfaction with their performance, their strong personality and ability of game management, with consequential improvements in the final perception of performance [11].

Previous research revealed that expert referees develop effective anticipatory strategies to improve the accuracy of their decision-making and the speed of intervention [12], show a better physical condition, a lower level of stress and make less penalty calls compared to lower-level referees [4, 10]. In fact, the best referees are more competent than their counterparts not only in terms of their decision making, but also in their more appropriate positioning on the field according to the flow of the game [13], allowing a better perception of players’ actions, the ball and the assistant referees [4, 12]. Also, it was reported that more experienced referees cooperate more effectively with assistant referees than less experienced referees, reducing the distance and the grey zones between them, and involving them more in the decisions [14]. In this line of reasoning, previous research indicated that 64% of all refereeing decisions are based on teamwork [4].

To assess a football referee match performance in a proper way, the Referee Convention guidelines of Europe’s football governing body (i.e., UEFA) mentioned that the assessment of a referee’s game performance should be accomplished by a Referee Match Observer (RMO) by means of a *live* observation in the stadium. In line with previous assumptions, the RMO should observe the match and register in an official assessment sheet the referee’s game score according to two main criteria of analysis (see Table 1 for details): i) game difficulty and game intervention; and ii) disciplinary actions and game management. The analysis comprises the evaluation of specific key performance indicators (KPIs) that allow a complementary evaluation of different competencies of the referee throughout the game [4]. Such competencies are aligned with the main characteristics of expert referees previously reported.

**Table 1:** Description of the various KPIs for each main criterion considered.

Variables	Description	Evaluation
**General evaluation**		
Referee’s game score	General performance of the referee	The score was between 10.00 (excellent performance) and <7.00 (very poor performance)
**Game difficulty and intervention**		
Difficulty	Difficulty and the incidences of the game	The score considered the critical incidents and the difficulty to management the game. E: Easy = "1", N: Normal = "2", D: Difficult = "3”
Personality	Personality of the referee during the game	The score considered the number of positive (Pos) = 1 and negative (Neg) = 2 game and players’ management during the game
Physical Condition_Position	Physical capacity of the referee	The score considered three groups of rating <4 = negative (Neg); 4 = neutral (N) >4 = positive (Pos)
Referee Teamwork	Teamwork between the referee and the assistant referees	The score considered the evaluation of three criteria and classify them in three groups <3 = negative; 3 = neutral; >3 = positive
**Disciplinary actions and game management**		
Disciplinary actions	Disciplinary actions during the game that include the cards displayed by the referee were registered	The score considered the number of yellow and red cards showed by the referee.
Application of the laws of the game P/N	Degree of application of the laws of the game	The score considered the number of positive (Pos) = 1 and negative (Neg) = 2 applications of the laws of the game
Disciplinary control and players management P/N	Disciplinary control of the referee during the game	The score considered the number of positive (Pos) = 1 and negative (Neg) = 2 interventions during the game

(Pos) Positive; (Neg) Negative

Despite of this process being used for a huge number of years in nearly all Football Associations (FAs), the relationship between the referee’s game score and the scores of the different KPIs is still unknown. In fact, to the best of our knowledge, such scale has never been validated or evaluated according to its impact on the final score of the referees. Also, previous research revealed variations in the reliability of the observed KPIs as well as in the final score [15]. Thus, despite of the impact of the game referee’s evaluation and game score in their upgrade, downgrade or maintenance in a given competitive level [16], further research is required with respect to the process of the assessment of football referees’ match performances. Particularly, there is a need to better understand the relationship and the contribution of each observed KPI on the referee’s game score.

Therefore, the purpose of this study was to evaluate the relationship between the best and worst referee’s game score and the KPIs evaluated on the RMO official assessment sheet. Specifically, our goal was to identify the contribution/weight of each KPI from game difficulty and intervention, and from disciplinary actions and game management to the final referee’s game score.

## Methods

### Participants

Thirty-four elite Referee Match Observers (RMOs) from the Portuguese FA voluntarily participated in the study (Age– 52.62 ± 8.95; Number of years as observer– 12.11 ± 6.81; Number of years as observer in 1st league– 7.18 ± 5.69). An informed and written consent was provided to the Portuguese FA Referee Council, as well as to all the participants before the beginning of the study. All participants were informed about the goal of the study and notified that they could withdraw from the study at any time. The study protocol followed the guidelines stated in the Declaration of Helsinki and was approved by the Local Ethics Committee.

### Data collection

Thirty-four games from the Portuguese main league 2016/2017 were analyzed in the stadium by an expert RMO and submitted to the Portuguese FA 24 hours after the end of the game. The RMO registered the analysis in an official assessment sheet that was produced by the Portuguese FA. The referee’s game score was registered in a scale between 0–10 (according to recommendations of Portuguese FA), and the evaluation of *game difficulty and intervention*, and *disciplinary actions and game management* registered according to the criteria for each specific KPI defined in the referee’s assessment score (see Table 1).

To access intra-individual reliability, a comparison between live and video analysis of the same match, with at least 8 weeks between observations and without any previous information about the game in analysis, was performed by all the participants. Due to non-normality of data, the referee’s assessment score was transformed in two steps: *a)* calculation of mean and standard deviation (SD) of the values of the untransformed variables; *b)* calculation of the Intraclass Correlation Coefficient (ICC) and the Bland and Altman graph, based on the variables after the transformation, with the correspondent 95% confidence limits (CL). Thresholds for ICC were <0.19, poor; 0.20–0.29, weak; 0.30–0.59, moderate; 0.60–0.79, substantial; and >0.80, very strong [17]. The intra-observer reliability revealed a substantial agreement between live and video observations (ICC = 0.73 [0.51–0.85], *p*< 0.001).

### Data analysis

From the official assessment sheet produced by each game, the referee’s game score was defined as the dependent variable and the KPIs from the two main dimensions game difficulty and game intervention, and disciplinary actions and game management, were identified as independent variables. A preliminary analysis revealed no missing values neither outliers. Moreover, for statistical purposes, a dummy variable of the referee’s game score was created, and the two groups were generated: i) Low Final Score (LFS)—games with a final score lower than the median final score, *n* = 19; ii); High Final Score (HFS)—games with a final game score higher than the median Final score, *n* = 15.

Subsequently, a descriptive analysis of the KPIs according to the final referee’s game score was conducted (continuous variables were described by means and standard deviation and categorical variables by absolute and relative frequencies). A multivariable binary logistic regression model was used to assess the relationship between the KPIs and the final referee’s game score. Only the variables whose univariate test was significant (*p*< .05) were selected for the model. Normality was checked using the Shapiro-Wilk test. Because the existence of non-normal distribution of data, the continuous KPI were analyzed by the *Mann-Whitney U test*. The relationships between referee’s game score and nominal KPI variables were analyzed through the Fisher’s exact test.

The Forward stepwise (Likelihood Ratio) selection method was considered, and the results were reported by odds ratio (OR) estimates and their 95% confidence intervals (CI). To evaluate the quality of the adjustment, the *Nagelkerke’s R*^*2*^ was used. Its interpretation as an effect size measure was made based on the following criteria: 0.02–0.13 small, 0.13–0.26 medium, and >0.26 large effect size. The model’s goodness of fit was assessed through the Hosmer-Lemeshow test and the area under the curve (AUC) through Receiver Operating Characteristic (ROC) was used to evaluate the discriminant capacity of the model. The ROC curve was performed using the predicted probabilities of each variable. All the statistical analyses were performed using SPSS 24.0 and statistical significance for rejecting the null hypothesis was set at *p*< .05.

## Results

Regarding the analysis of the dimension *game difficulty and game intervention*, results revealed significant differences between LFS and HFS for the KPIs *personality* and *referee teamwork* (*p*< .05). The LFS revealed lower number of positive interventions with respect to *personality* and lower positive actions of *referee teamwork* than HFS (see Table 2).

**Table 2:** Results of the KPIs for difficulty and intervention for both low final score and high final score.

		LFS	HFS	*p-value #1*
Difficulty 1st	E	0	0	1
	N	18 (94.7%)	14 (93.3%)	
	D	1 (5.3%)	1 (6.7%)	
Difficulty 2st	E	0	0	0.672
	N	16 (84.2%)	11 (73.3%)	
	D	3 (15.8%)	4 (26.7%)	
Personality	Pos	11(57.9%)	15 (100%)	**0.005** *
	Neg	8 (42.1%)	0	
Physical Condition_Position	Neg	5 (26.3%)	4 (26.7%)	0.413
	Neu	10 (52.6%)	5 (33.3%)	
	Pos	4 (21.1%)	6 (40%)	
Referee Teamwork	Neg	4 (21.1%)	1 (6.7%)	**0.007** *
	Neu	13 (68.4%)	5 (33.3%)	
	Pos	2 (10.5%)	9 (60%)	

#1 –Exact Fisher test

* p<0.01; (E) Easy; (N) Neutral; (D) Difficult; (Pos) Positive; (Neg) Negative; (Neu).

The analysis of the dimension *disciplinary actions and game management* revealed significant differences between LFS and HFS for KPIs *negative application of the laws of the game* and *negative disciplinary control and players management variables* (*p*< .05). The LFS revealed higher number of *negative application of the laws of the game* and also a *higher number of negative disciplinary contro*l and *players’ management* (see Table 3).

**Table 3:** Results of the KPIs for criteria disciplinary actions and game management for both low final score and high final score.

Variable	LFS (Mean ± SD)	HFS (Mean ± SD)	*p-value#2*
Disciplinary actions	7±2.03	6.93±2.12	0.876
Application of the laws of the game Pos	1.747±1.22	2.13±1.13	0.127
Application of the laws of the game Neg	1.37±1.21	0.27±0.46	**0.002** *
Disciplinary control and players management Pos	0.53±0.91	0.60±0.83	0.598
Disciplinary control and players management Neg	1.16±0.83	0.53±0.52	**0.021** **

#2 –U Mann-Whitney test

* p<0.01

** p<0.05

In the multivariable binary logistic regression model, the independent KPIs *personality and disciplinary control* and *negative players management* were excluded from the model (*p*>.05). The overall model presented a well-fitting value (p_Hosmer-Lemeshow_> .05), a good correct global classification (79.4%) and its discriminant capacity was also quite good with the AUC ranging between .788 –.998, with a 95% confidence level. The model accounts for 60.4% of the explanation of the referee’s game score (*Negelkerke R*^*2*^ = 0.604), corresponding to a large effect size (see Table 4).

**Table 4:** Estimated regression coefficients from binary logistic regression analysis, only for significant variables (Final game score as dependent variable).

Variables	*B*	OR (CI 95%)	p	AUC (CI 95%)
Application of the laws of the game Neg	-1.92	0.15 (0.29–0.750)	.021	.893 (.788 - .998)
Referee Teamwork				
Negative^b^		1		
Neutral	1.21	3.35 (0.22–50.68)	.383	
Positive	3.83	45.84 (1.67–1258.35)	.024	
Nagelkerke R^2 = 0.604	Hosmer-Lemeshow test (p = .961)		Correct global classification = 79.4%	

Note. OR—Odds ratio; ^b^ Reference category; AUC—Area under the ROC curve

The results revealed that for each negative application of the laws of the game, the chances to achieve a HFS decreased by 85% (*OR* = 0.15, *CI*_*95%*_ = [0.29–0.75]). Also, the analysis of the referee teamwork revealed that a referee with a neutral evaluation has three times more chances to achieve a HFS than a referee with a negative evaluation (*OR* = 3.35, *CI*_*95%*_ = [0.22–50.68]). A referee with a positive evaluation has forty-six times more chances to achieve a HFS than a referee with a negative evaluation (*OR* = 45.84, *CI*_*95%*_ = [1.67–1258.35]) (see Table 4).

## Discussion

The purpose of this study was to evaluate the contribution/weight of each KPI from game difficulty and intervention, and from disciplinary actions and game management, to the final referee’s game score. The results highlighted that two variables of each category clearly distinguish the best to the worst referees based on the referee’s game performance assessment. From the category game dificulty and intervention, the variables personality and referee teamwork were the unique variables that distinguish LFS and HFS referees. Interestingly, in the category disciplinary actions and game management, the variables negative application of the laws of the game, and negative disciplinary control and player management were the unique variables that distinguish LFS and HFS referees. The results from multivariable binary logistic regression model revealed that the KPIs referee teamwork and negative application of the laws of the game and were the ones that significantly contributed to the final game referee’s assessment score. The model accounts for 60.4% of the explanation of the referee’s game score.

### Characterization of game LFS and HFS referee’s assessment

The development of referee excellence and skills are strongly dominated by the existence of evaluation/rating processes, being of central preponderance for their upgrade, downgrade or maintenance in a certain competitive level [16]. Here, it was possible to highlight the skills/performance indicators that best distinguish LFS and HFS referees. Regarding difficulty and intervention, the HFS show better performance in terms of the personality as well as in the teamwork level compared to the LFS. Thus, the elite referees that revealed better performance seem to be able, probably due to a better capacity for cooperation with the assistant referees, to judge and decide with higher accuracy, adequacy and consistency according to the game demands [10, 14]. Thus, for their match preparation, referees should be aware about the strategies of leadership assumed over the game according to the game context, as well as the capacity of communication and teamwork with the assistant referees.

Regarding the results of the disciplinary criteria and the management of the game, the HFS revealed lower errors (negative) in terms of decision making with respect to the application of the laws of the game, as well as in the disciplinary control and management of players. Each decision and intervention of the referee is critical during the game and may dramatically affect the result of the match [18]. Thus, particularly the negative application of the laws of the game and the negative disciplinary control and management of players should be avoided to improve referees’ performances. Based on these results, it is possible to confirm previous assumptions that the best referees are the ones that demonstrate greater ability to correctly apply the disciplinary criteria described in the laws of the game [4].

### Key performance indicators of the referee’s game assessment score

The referee’s game score incorporates different dimensions of a referee’s performance [19]. In general, the analysis comprises the definition of the final score of referee assessment but also the evaluation of the KPIs such as the technical and disciplinary decisions, the difficulty of the game, the positioning, as well as the capacity of game management and collaboration with assistant referees [10, 20]. Despite this process being used for a large number of years in nearly all Football Associations (FAs), the relation between the final score of referee assessment and the scores of the different criteria evaluated remained somewhat unclear.

Previous research proposed that the performance of elite referees implicate a continuous analysis of the game that depends on a proper level of interaction with assistant referees [20]. Therefore, the capacity of the referee to develop teamwork, share information and decisions with assistant referees is essential to improve the referee’s performance [1]. A referee with a negative score on the KPI teamwork, has three times less chance to be in the HFS group, than a referee who scores neutral on teamwork. Likewise, a referee who gets a negative score on the KPI teamwork has 46 times less chance to be in the HFS group, compared to the referee with a positive score. Such result is particularly important for the future, due to the integration of the technological aids such as the communication system, goalline technology and the Video Assistant Referee. During refereeing courses, specific work should be promoted to develop the teamwork between the referees and assistant referees not only in terms of communication but also in terms of decision-making, coherence of interventions and trust for the correct application of the laws of the game and also game management.

As mentioned previously, football referees are not only required to make accurate decisions and to apply in a consistent way the disciplinary criteria [2] based on the laws of the game, but also to ensure an appropriate management of the game [3, 8]. In other words, referee with a negative score in the application of the laws of the game, more specifically in the disciplinary action and game management, has eighty-five times less change to be in the HFS group, compared to the referee with a positive score. In fact, Collina [21] considered that more than the knowledge about the rules of the game, the best referees should use such knowledge to maintain consistency on the application of the disciplinary aspects according to the game dynamics and the contextual factors that interfere with it [4, 15]. Such result is a great contribution for the preparation of novice referees reinforcing the need to promote the acquisition of knowledge of the game (i.e., that sustain the capacity of referees to apply the disciplinary criteria in relation to the game dynamics) and not the knowledge about the game per se (i.e., that allows the description of the game rules and the consequent disciplinary criteria without any reference to the context) [22]. Further research should be developed to understand if not only the number, but also the nature of disciplinary actions may influence the referee’s game score.

The results of this study build on this lack of knowledge about the use of such assessment score to reveal that the KPIs referee teamwork and negative application of the laws of the game explained 60.4% of the referee’s game score, with a very good discriminant capacity of the model. Probably the model cannopt explains high percentage of game score due to the lack of objective criteria for the calculation of the final referee’s game score.

In fact, the referee’s game score was not calculated based on the scores atributed to each variable in each category, but based on a recommendation from the Referee Convention guidelines of UEFA. Thus, for a further relationship between the overall variables and the final referee’s game score, a more reliable method is required with a clear ponderation of the weight of each variable for the calculation of the final score.

Despite the fact that this study stands as the first attempt to gain a further understanding of the referee’s game score according to the contribution of each category for the computation of the final score, there are some limitations that should be declared. First, data collection occurred in a single country and during only one season. Therefore, in a certain way, it is representative of a quite specific context. Also, the relationship between the KPIs analyzed and the disciplinary and game management events such as, for example, a penalty kick or a display of a correct or incorrect red card were not considered and should certainly be considered in future studies. At the end, this study should be developed using recent data that considered the use of data from the new technological aids such as the Video Assistant Referee.

## Conclusion

In conclusion, this study suggests that the KPIs scored by the RMOs contributed in a different way to the referee’s game score. The model accounts for 60.4% of the variation of the referee’s game score. However, the referees with the best referee’s game score still revealed a higher number of positive interventions regarding personality and lower number of negative disciplinary control and players’ management compared to the referees with the worst referee’s game score. The results did not reveal any difference for the variables game difficulty, physical condition, position, disciplinary actions, positive application of the laws of the game and positive disciplinary control and players management. Based on this study, relevant stakeholders such as Football Associations (FAs) can take advantage of these results to improve the methods of match assessment paying particular attention to the critical KPIs in an appropriate way. Further research is required to develop a more reliable method that objectively considers the weight of each variable in an appropriate way to determine the final score.
